# Clinicopathological and Preclinical Patient-Derived Model Studies Define High Expression of NRN1 as a Diagnostic and Therapeutic Target for Clear Cell Renal Cell Carcinoma

**DOI:** 10.3389/fonc.2021.758503

**Published:** 2021-11-03

**Authors:** Shuhei Kamada, Kazuhiro Ikeda, Takashi Suzuki, Wataru Sato, Sachi Kitayama, Satoru Kawakami, Tomohiko Ichikawa, Kuniko Horie, Satoshi Inoue

**Affiliations:** ^1^ Division of Systems Medicine & Gene Therapy, Saitama Medical University, Saitama, Japan; ^2^ Department of Urology, Graduate School of Medicine, Chiba University, Chiba, Japan; ^3^ Department of Pathology and Histotechnology, Tohoku University Graduate School of Medicine, Miyagi, Japan; ^4^ Department of Urology, Saitama Medical Center, Saitama Medical University, Saitama, Japan; ^5^ Department of Systems Aging Science and Medicine, Tokyo Metropolitan Institute of Gerontology, Tokyo, Japan

**Keywords:** renal cell carcinoma, patient-derived cancer cell (PDC), patient-derived xenograft, cancer stem-like cell, cancer stemness, spheroid, neuritin 1, C-X-C chemokine receptor type 4

## Abstract

**Background:**

Acquired therapeutic resistance and metastasis/recurrence remain significant challenge in advance renal cell carcinoma (RCC), thus the establishment of patient-derived cancer models may provide a clue to assess the problem. We recently characterized that neuritogenesis-related protein neuritin 1 (NRN1) functions as an oncogene in testicular germ cell tumor. This study aims to elucidate the role of NRN1 in RCC.

**Methods:**

NRN1 expression in clinical RCC specimens was analyzed based on immunohistochemistry. *NRN1*-associated genes in RCC were screened by the RNA-sequencing dataset from The Cancer Genome Atlas (TCGA). RCC patient-derived cancer cell (RCC-PDC) spheroid cultures were established and their viabilities were evaluated under the condition of gene silencing/overexpression. The therapeutic effect of NRN1-specific siRNA was evaluated in RCC-PDC xenograft models.

**Results:**

NRN1 immunoreactivity was positively associated with shorter overall survival in RCC patients. In TCGA RCC RNA-sequencing dataset, *C-X-C chemokine receptor type 4* (*CXCR4*), a prognostic and stemness-related factor in RCC, is a gene whose expression is substantially correlated with *NRN1* expression. Gain- and loss-of-function studies in RCC-PDC spheroid cultures revealed that *NRN1* significantly promotes cell viability along with the upregulation of *CXCR4*. The NRN1-specific siRNA injection significantly suppressed the proliferation of RCC-PDC-derived xenograft tumors, in which *CXCR4* expression is significantly repressed.

**Conclusion:**

NRN1 can be a potential diagnostic and therapeutic target in RCC as analyzed by preclinical patient-derived cancer models and clinicopathological studies.

## Introduction

Renal cell carcinoma (RCC) is the most common type of kidney cancer in adults, with >400,000 new cases diagnosed worldwide in 2018 (1, 2). Patients with resectable RCC have relatively good prognosis with a 5-year survival rate of >90%, whereas those with recurrent or metastatic RCC still have a poor prognosis with a 5-year survival rate of 10–20% (3). New therapeutic strategies including immune checkpoint inhibitors improve prognosis of RCC patients nowadays; yet not all patients respond to these therapies and positive responses are usually achieved in a minority of cases (4, 5). Thus, more effective RCC strategy to all RCC patients remains to be developed.

Recent advance in cancer research technology has provided useful platforms to dissect potential diagnostic and therapeutic targets. In particular, patient-derived cancer models are applied to preclinical tests for drug screening. We have established patient-derived cancer cell (PDC) spheroid culture systems. The three-dimensional culture technology is useful to preferentially enrich cancer stem-like cells (CSCs) with self-renewal, a cell population often responsible for tumor recurrence/metastasis and therapeutic resistance (6–11). PDC system also has a particular advantage that can be applied to establish *in vivo* PDC-derived xenograft (PDCX) models (12–14).

We recently demonstrated that neuritogenesis-related protein neuritin 1 (NRN1) can be a potential therapeutic target in testicular germ cell tumor (TGCT) under the regulation by HIF1α (12). Because NRN1 expression is positively associated with the proliferation of patient-derived TGCT spheroid cultures with cancer stemness features, we here extend our question whether NRN1 contributes to the proliferation of RCC cells, particularly in clear cell carcinoma where HIF1α signaling is predominantly activated due to mutational inactivation of VHL. While NRN1 is a glycosylphoshatidylinositol-anchored protein and involved in neuritogenesis (15), NRN1 expression is hypoxia-inducible and its high mRNA expression was observed in a restricted number of tumor cells around perinecrotic regions of a case of conventional RCC (16).

In the present study, based on our findings and previous literature, we aimed to characterize whether NRN1 contributes to RCC biology, particularly to cancer stemness in ccRCC. NRN1 plays a tumor-promoting role in RCC. PDC/PDCX-based and pathological analyses of RCC uncovered that NRN1 and its co-expressing molecule CXCR4 are positively associated with RCC patient prognosis and their silencing substantially suppresses PDC viability and PDCX tumor growth. Our findings will provide a potential basis for the development of alternative diagnostic and therapeutic options for patients with RCC.

## Materials and Methods

### Clinical Data Collection and Patient Selection

A cohort of 100 patients with clear cell RCC (ccRCC) diagnosed at the Saitama Medical Center between 2008 and 2019 were retrospectively analyzed. We evaluated the overall survival of the patients from the time of their first visit. Patient characteristics are shown in **Table 1**. Patients who did not agree to participate in the study were excluded. The clinical parameters whose values were not available were excluded from the statistical analysis to compare the patient characteristics. Pathological findings were classified according to the Japanese General Rules for Clinical and Pathological Studies on Renal Cell Carcinoma (17). The Institutional Review Board of the Saitama Medical Center, Saitama Medical University, approved the clinical protocols (No. 117 and No. 2308).

**Table 1:** Association between NRN1 immunoreactivity and clinicopathological factors in 100 clear cell RCCs.

	NRN1 immunoreactivity		*P* value
	High (*n* = 21)	Low (*n* = 79)	
Age (years, median 67)			
≥ 67	13	41	
< 67	8	38	0.47
Gender			
Male	15	54	
Female	6	25	1.0
Stage			
I	10	43	
II	0	11	
III	3	11	
IV	8	14	0.10
Pathological T factor (pT)			
pT1-2	14	62	
pT3-4	7	17	0.26
Lymph node metastasis			
Positive	3	2	
Negative	18	77	*0.061*
Distant metastasis			
Positive	8	13	
Negative	14	65	*0.072*
Grade (Furhman)			
1-2	14	54	
3-4	7	25	1.0
IMDC risk group classification			
Favorable	6	35	
Intermediate	10	43	
Poor	5	1	**0.0027**
Hb (g dl^-1^, median M: 13.2, F: 12.2)			
≥ LLN ^a^	10	44	
< LLN	11	35	0.62
Corrected serum calcium ^b^ (mg dl^-1^, median 9.3)			
≥ 10	9	11	
< 10	12	68	**0.011**
LDH (g dl^-1^, median 184)			
≥ 1.5 × ULN ^c^	1	1	
< 1.5 × ULN	20	78	0.38
CRP (mg dl^-1^, median 0.20)			
≥ 0.20	17	41	
< 0.20	4	38	**0.024**

P value < 0.05 and 0.05 ≤ P value < 0.10 were significant (in bold) and borderline significant (in italics), respectively.

a LLN of Hb are 13.5 and 11.3 g/dL for male and female, respectively.

b Corrected serum calcium (mg dl^-1^) = measured total calcium (mg dl^-1^) + 0.8 (4.0 - serum albumin (g dl^-1^).

c ULN of LDH is 245 U l^-1^.

IMDC, International Metastatic RCC Database Consortium; Hb, hemoglobin; LLN, lower limit of normal; ULN, upper limit of normal; LDH, lactate dehydrogenase; CRP, C-reactive protein.

The Cancer Genome Atlas (TCGA) RCC dataset retrieved from The Human Protein Atlas (https://www.proteinatlas.org/) was used for prognostic analysis for *NRN1* and *CXCR4* mRNA. For co-expression analysis, mRNA expression data in TCGA ccRCC RNA-sequencing (RNA-seq) dataset were retrieved from cBioPortal (https://www.cbioportal.org/). Gene ontology (GO) analysis was performed using The Database for Annotation, Visualization and Integrated Discovery (DAVID) (https://david.ncifcrf.gov/tools.jsp).

### Patient-Derived RCC Spheroid Cultures and Cell Lines

Patient-derived ccRCC spheroid cultures were established from primary tumors of patients with RCC after obtaining informed consent at the Saitama Medical Center. Tumor samples were processed and cultured as three-dimensional spheroids as described previously (13). Characteristics of the patient PDCs are summarized in **Supplementary Table 1**. The Ethics Committee of the Saitama Medical Center, Saitama Medical University, approved all procedures (No. 1363-IV).

### cDNA Synthesis and Quantitative Reverse-Transcription Polymerase Chain Reaction

RNA extraction, cDNA synthesis, and qRT-PCR were performed as described previously (13). β-Actin (ACTB) was used as the internal control. All qRT-PCR primers used in this study are listed in **Supplementary Table 2**.

### siRNA, Expression Vector, and Transfection

siRNAs targeting *NRN1* (siNRN1 #1 and #2) and control siRNA (siControl) were purchased from RNAi Inc. and transfected into PDCs with RNAiMAX reagent (Thermo Fisher Scientific) according to the manufacturer’s instructions. The siRNA sequences used are shown in **Supplementary Table 3**. Flag-tagged NRN1 cDNA was subcloned into pcDNA3 (Invitrogen) and transfected into PDCs with Lipofectamine 3000 reagent (Thermo Fisher Scientific) according to the manufacturer’s instructions.

### Cell Viability Assay for Spheroid Cultures

Cell viability was assessed using the CellTiter-Glo 3D Assay (Promega) on day 3 after plating and transfection.

### Western Blotting

RCC-PDC1 cells transfected with siRNAs or expression vectors were lysed in RIPA buffer (50 mM Tris-HCl, pH 7.5, 150 mM NaCl, 0.1% SDS, 1% Triton X-100, 1% sodium deoxycholate, 1 mM PMSF, 1 μg/mL aprotinin) and the plasma membrane fraction was purified by centrifugation at 19,100*g* for 20 min at 4°C. Proteins were separated with SDS-PAGE and blotted on PVDF membrane, followed by reactions with anti-CXCR4 antibody (GTX22074, GeneTex) or anti-β-actin antibody (AC-74, SigmaAldrich) for loading control.

### Immunohistochemistry

Formalin-fixed tissues and PDCs resuspended in iPGell (Genostaff) were embedded in paraffin and sectioned as previously described (13). A Histofine kit (Nichirei) based on the streptavidin-biotin amplification method was used for immunohistochemical analyses of NRN1 (anti-NRN1 antibody PRS4101, Sigma-Aldrich) and CXCR4 (anti-CXCR4 antibody GTX22074, GeneTex). Specialized pathologists evaluated the percentage of immune-positive tumor cells. Immunoreactivity of NRN1 and CXCR4 was detected in the cytoplasm of RCC cells and considered high when the cases had more than 10% of the positive carcinoma cells.

### Animal Experiments

All animal experiments were approved by the Animal Care and Use Committee of Saitama Medical University, and carried out in accordance with the Guidelines and Regulations for the Care and Use of Experimental Animals of Saitama Medical University. For the xenograft model, 1.5 × 10^6^ RCC-PDCs were suspended in 150 μl of medium containing 50% Matrigel (BD Biosciences, San Diego, CA) and subcutaneously injected into the flank of male NOG mice (*In-Vivo* Science, Washington, DC) or male nude mice (BALB/cAJcl-nu/nu) as described previously (13). The RCC-PDC1-derived tumor generated in nude mice was cut into 2-mm cubes and inoculated into the flanks of 8-week-old nude mice. The tumors were measured in two-dimension using micrometer calipers, and the tumor volume was estimated according to the formula: 0.5 × ((smallest diameter)^2^ × (longest diameter)). When the tumor volume exceeded 150 mm^3^, mice were randomly assigned to one of two groups: siNRN1 #1- and siControl-administered groups (*n* = 5, each group). siRNA duplexes (5 μg) were mixed with 4 μl GeneSilencer reagent (Gene Therapy System, San Diego, CA), dissolved in 50 μl Dulbecco’s modified Eagle’s medium, and then directly injected into the tumors twice weekly. The results were represented as mean ± SD. Student’s *t*-test was used for statistical analysis. At the end point of the experiment (3 weeks after siRNA administration), the tumors were dissected from the mice.

### Statistical Analysis

Clinical data were analyzed using the log-rank test for the Kaplan-Meier method and Fisher’s exact test for patient characteristics. Experimental data were analyzed using a two-sided Student’s *t*-test for pairwise comparisons. For multiple comparisons, a two-way ANOVA test was used. Univariate and multivariate analyses were performed using the Cox proportional hazard model. Overall survival curves were generated using the Kaplan-Meier method, and statistical significance was determined using the log-rank test. Statistical computations were carried out using EZR software (version 1.54) (18).

## Results

### NRN1 Expression is Correlated With Poor Prognosis in Patients With RCC

**Figure 1:**
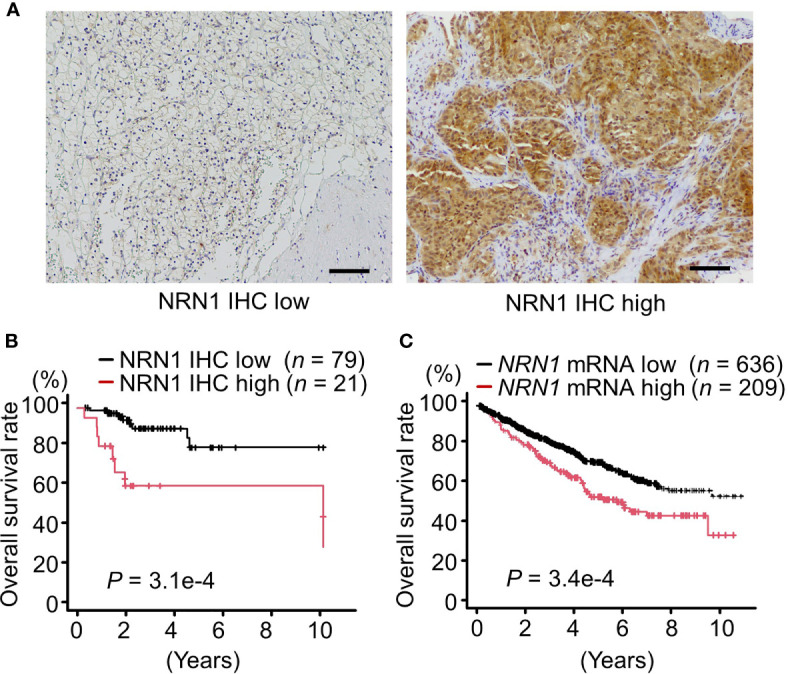
NRN1 is a poor prognostic factor for patients with RCC. **(A)** Representative immunohistochemistry (IHC) staining of low (left panel) and high (right panel) expression of NRN1 in RCC tissue sections. Scale bars, 100 µm. **(B)** Overall survival of 100 patients with clear cell RCC (ccRCC) of low or high NRN1 immunoreactivity was analyzed by Kaplan-Meier method. Statistical significance was evaluated by log-rank test. **(C)** Overall survival of RCC patients with low or high *NRN1* mRNA levels were analyzed by Kaplan-Meier method. *NRN1* expression data in TCGA RCC dataset were retrieved from The Human Protein Atlas (*n* = 845). High and low *NRN1* expressions were determined at an expression cut off level as 6.6 fragments per kilobase of exon per million mapped fragments (FPKM) (expression level range: 0.1-204.2, expression median level: 2.9 FPKM, *P* = 3.4e-4). Statistical significance was evaluated by log-rank test.

To assess the clinical significance of NRN1 in RCC, we performed immunohistochemical (IHC) analysis of NRN1 in ccRCC tumor samples obtained from distinct patients. We determined that 21 of 100 tumor tissues exhibited high NRN1 IHC staining in the cytoplasm of cancer cells (**Figure 1A**). Kaplan-Meier analysis of the 100 cases showed that the patients with high NRN1 IHC staining showed significant shorter overall survival rate than those with low NRN1 IHC staining (*P* = 3.1e-4) (**Figure 1B**). In clinicopathological analysis, NRN1 immunoreactivity was significantly correlated with corrected serum calcium (*P* = 0.011) and C-reactive protein (CRP) (*P* = 0.024) (**Table 1**). NRN1 immunoreactivity was also associated with the International Metastatic RCC Database Consortium (IMDC) risk group classification, which is used to stratify patients with metastatic RCC (*P* = 0.0027). Furthermore, univariate and multivariate analyses of overall survival showed that NRN1 immunoreactivity was an independent prognostic factor for RCC (*P* = 0.0012 and 0.036, respectively) (**Table 2**). We also presented representative IHC images of NRN1 with disease stages (I-IV) in neoplastic and paired adjacent non-neoplastic tissues in **Supplementary Figure 1**. These NRN1 IHC images and the association study of **Table 1** may indicate that there is a positive tendency for the correlation between NRN1 immunoreactivity and stages. Grades were not basically associated with NRN1 immunoreactivity as shown in **Table 1**. In addition, high NRN1 immunoreactivities were found in neoplastic areas at stages III and IV compared with their paired corresponding non-neoplastic areas. Of 100 paired analyses, high immunoreactivities were detected in 21 neoplastic areas whereas not in non-neoplastic areas (*P* < 1.0e-4 by McNemar’s test). In RNA-seq dataset of TCGA RCC cohort (*n* = 845), patients with high *NRN1* mRNA levels had shorter overall survival rate than those with low *NRN1* levels (expression level range: 0.1-204.2 FPKM, expression median level: 2.9, expression cut off level 6.6 FPKM, *P* = 3.4e-4) (**Figure 1C**). *NRN1* mRNA levels in stages III and IV RCC tumors significantly higher in those in stages I and II RCC tumors (*P* = 5.4e-6) (**Supplementary Figure 2A**). Moreover, we examined RCC-subtype dependent NRN1 expressions and prognostic values based on the TCGA datasets for ccRCC (KIRC), papillary RCC (KIRP), and chromophobe RCC (KICH) through the Human Protein Atlas website. As shown in **Supplementary Figure 3**, KIRC tissues expressed higher levels of NRN1 compared with KIRP and KICH tissues. In addition, high NRN1 expression was significantly associated with poor survival in patients with KIRC and KIRP. These results suggest that NRN1 may play an oncogenic role in ccRCC and renal papillary cell carcinoma at least.

**Table 2:** Univariate and multivariate analyses of overall survival in 100 clear cell RCC patients.

Variables	Univariate	Multivariate	
	*P* value	*P* value	Relative risk (95% CI)
Corrected serum calcium^a^	**5.0e-6^†^**	0.22	2.6 (0.57-12)
(≥ 10 mg dl^-1^/< 10 mg dl^-1^)			
LDH	**1.1e-4^†^**	**0.0057**	22 (2.5-201)
(≥ 1.5 × ULM ^b^/< 1.5 × ULM)			
Distant metastasis	**1.6e-4^†^**	**0.021**	5.1 (1.3-20)
(positive/negative)			
NRN1 immunoreactivity	**0.0012^†^**	**0.036**	4.2 (1.1-16)
(High/Low)			
Pathological T factor (pT)	**0.0022^†^**	0.49	1.6 (0.42-6.1)
(pT3-4/pT1-2)			
Hb	**0.021^†^**	0.41	0.50 (0.094-2.6)
(≥ LLN ^c^/< LLN)			
CRP	*0.066* **^†^**	0.83	0.80 (0.11-5.8)
(≥ 0.20 mg dl^-1^/< 0.20 mg dl^-1^)			
Lymph node metastasis	*0.079* **^†^**	0.87	0.86 (0.14-5.3)
(positive/negative)			
Age	0.37		
(≥ 67/< 67)			
Grade	0.66		
(3,4/1,2)			
Gender	0.82		
(male/female)			

Statistical analysis was evaluated by a proportional hazard model (Cox).

P value < 0.05 and 0.05 ≤ P value < 0.10 were significant (in bold) and borderline significant (in italics), respectively.

95% CI, 95% confidence interval.

^†^Significant (P < 0.05) and borderline significant (0.05 ≤ P value < 0.10) values were examined in the multivariate analyses in this study.

a Corrected serum calcium (mg dl^-1^) = measured total calcium (mg dl^-1^) + 0.8 (4.0 - serum albumin (g dl^-1^).

b ULN of LDH is 245 U l^-1^.

c LLN of Hb are 13.5 and 11.3 g/dL for male and female, respectively.

Hb, hemoglobin; LLN, lower limit of normal; ULN, upper limit of normal; LDH, lactate dehydrogenase; CRP, C-reactive protein.

### CXCR4 Is a Stemness-Related Gene Co-Expressed With NRN1 in RCC and Its Expression Is a Prognostic Biomarker in RCC

We next explored genes co-expressed with *NRN1* in ccRCC tumors. Because we previously identified that NRN1 was abundantly expressed in patient-derived testicular germ cell tumor spheroid cultures with the enrichment of cancer stemness characteristics, we particularly examined the co-expression of NRN1 with stemness-related genes in RCC. In TCGA PanCancer Atlas RNA-seq dataset of ccRCC cohort (*n* = 352), we found that *C-X-C chemokine receptor type 4* (*CXCR4*) is a stemness-related gene whose expression is substantially correlated with *NRN1* expression (Spearman’s correlation coefficient = 0.263, q-value 9.83e-6). In terms of other prototypic stemness-related genes such as CD44, OCT3/4, and SOX2, Spearman’s correlation coefficients for co-expression were <0.2. Based on the TCGA PanCancer Atlas RNA-seq dataset of ccRCC cohort, we extracted 1,974 and 3,265 genes co-expressed with *NRN1* and *CXCR4*, respectively, with Spearmen’s correlation coefficient ≥0.2 and determined 941 common genes as the overlap of genes co-expressed with both *NRN1* and *CXCR4* (**Supplementary Figure 4**). Among the top 10 identified Gene Ontology (GO) Terms using The Database for Annotation, Visualization and Integrated Discovery (DAVID), pathways related to such as extracellular matrix, collagen catabolic process, collagen fibril organization, angiogenesis, and cell adhesion were identified.

We next examined the correlation of CXCR4 expression with RCC patient prognosis in the 100 ccRCC tumor specimens as described above. Prior to immunohistochemical study, the CXCR4 antibody was used in Western blotting where CXCR4 protein levels were decreased and increased by NRN1 silencing and overexpression in RCC-PDC1 cells, respectively (**Supplementary Figures 5A, B**). We determined 25 of 100 tumor tissues showed high CXCR4 IHC staining in the cytoplasm of cancer cells (**Supplementary Figure 5C**). Kaplan-Meier analysis of the 100 cases showed that the patients with high CXCR4 IHC staining showed significant shorter overall survival rate than those with low CXCR4 IHC staining (*P* = 3.0e-5) (**Supplementary Figure 5D**). In RNA-seq dataset of TCGA RCC cohort (*n* = 845), patients with high *CXCR4* mRNA levels had shorter overall survival rate than those with low *CXCR4* levels (expression level range: 2.1-372.0 FPKM, expression median level: 61.8, expression cut off level: 99.5 FPKM, *P* = 1.1e-5) (**Supplementary Figure 5E**). Similar to *NRN1* expression, *CXCR4* mRNA levels in stages III and IV RCC tumors significantly higher in those in stages I and II RCC tumors (*P* = 1.3e-6) (**Supplementary Figure 2B**).

### NRN1 and CXCR4 Expression in Patient-Derived RCC Models

**Figure 2:**
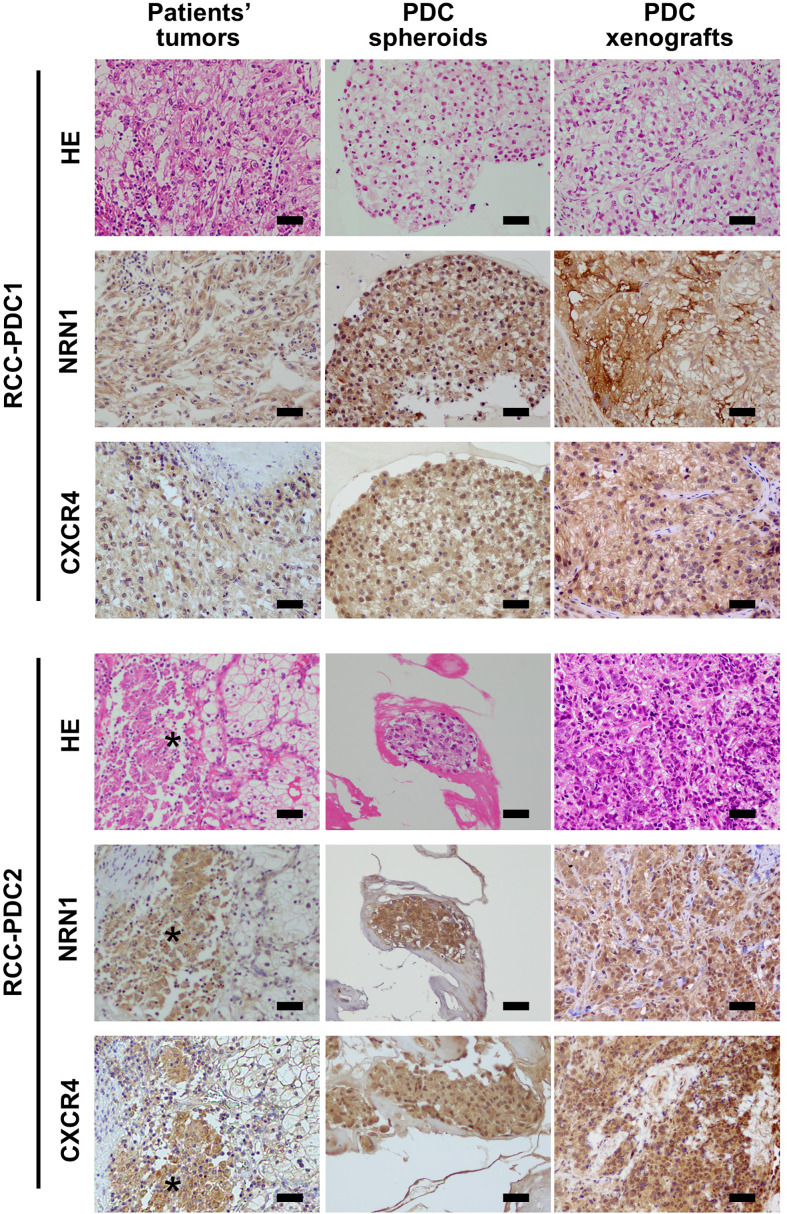
Histological analysis of RCC primary tumors, and their patient-derived cancer models. Hematoxylin and eosin (HE) staining, NRN1 and CXCR4 immunohistochemistry of primary tumor specimens, corresponding patient-derived cancer cell (PDC) spheroid cultures, and their xenograft tumors. Star represents ccRCC tumor portion with eosinophilic cytoplasm. Scale bars, 50 µm.

To investigate the roles of NRN1 and CXCR4 in RCC, we established RCC-PDC spheroid cultures from 2 distinct patients with ccRCC, RCC-PDC1 and RCC-PDC2. Transplantation of RCC-PDC1 and RCC-PDC2 into the flanks of immunocompromised male NOG mice could successfully generate PDC-originated xenograft (PDCX) tumors. Hematoxylin and eosin (HE) staining and NRN1 immunostaining showed that RCC-PDC1 spheroid culture and its PDCX tumors recapitulated the morphological and immunohistological features of ccRCC (**Figure 2**). In the case of RCC-PDC2, the original patient tumor contained a tissue portion of ccRCC cells with eosinophilic cytoplasm and high NRN1/CXCR4 IHC staining, which feature was recapitulated in the established PDC spheroid culture and its PDCX tumor (**Figure 2**).

### NRN1 and CXCR4 Contribute to RCC-PDC Viability

**Figure 3:**
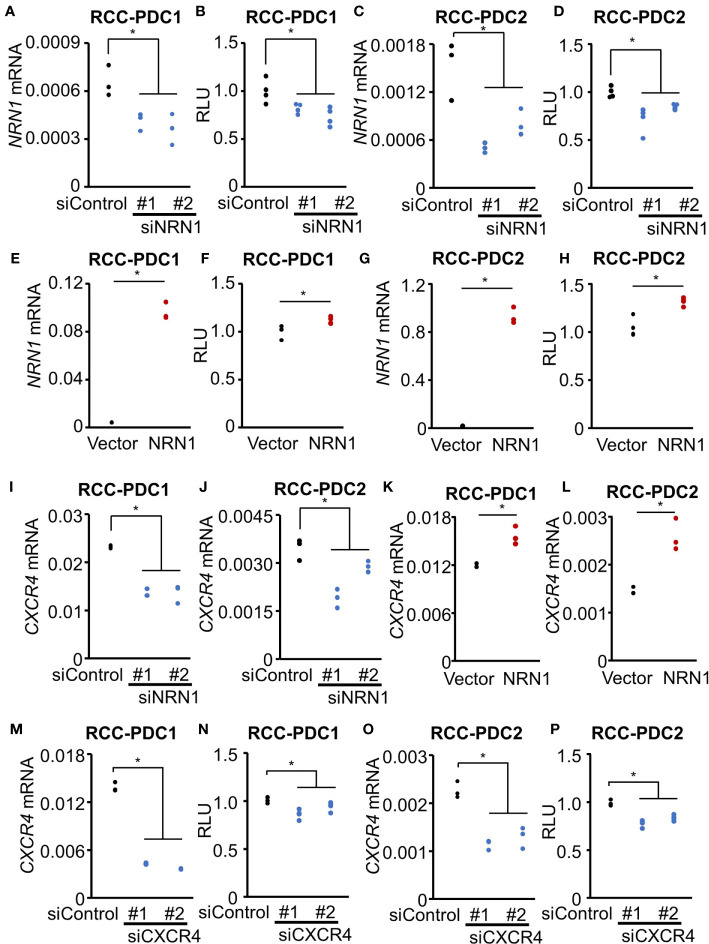
NRN1 and CXCR4 promote RCC-PDC viability. **(A–H)** NRN1 silencing decreases whereas overexpression increases PDC viability. RCC-PDC1/2 spheroid cultures were transfected with NRN1 (siNRN1 #1 and #2) or control (siControl) siRNAs **(A–D)**, and NRN1 or control expression vector **(E–H)**. NRN1 mRNA levels were analyzed by qRT-PCR **(A, C, E, G)** (*n* = 3) and spheroid growth was estimated by cell viability assay based on ATP quantification in cell lysates **(B, D, F, H)** (*n* = 4). Data are shown as means ± SD. *P < 0.05 by two-sided Student’s *t*-test. **(I–L)** NRN1 silencing decreases whereas overexpression increases CXCR4 expression. RCC-PDC1/2 were transfected with siRNAs: siNRN1 #1, #2, or siControl **(I, J)**, or expression vectors (NRN1 or control vector) **(K, L)**. **(M–P)** CXCR4 silencing represses PDC viability. RCC-PDC1 and 2 were transfected with siRNAs targeting CXCR4 (siCXCR4 #1 and #2) or siControl. CXCR4 mRNA levels were analyzed by qRT-PCR **(M, O)** and spheroid growth was estimated by cell viability assay **(N, P)**. Data are shown as means ± SD, *n* = 3. *P < 0.05 by two-sided Student’s *t*-test.

We next questioned whether NRN1 and CXCR4 play roles in RCC cell proliferation. We evaluated the effect of *NRN1* knockdown on RCC-PDC viability by transfecting *NRN1*-specific siRNAs siNRN1 #1 and #2. These siRNAs significantly repressed *NRN1* expression levels in PDCs compared with the control siRNA (siControl) (**Figures 3A, C** **)**, and impaired the viabilities of RCC-PDC1 and -PDC2 spheroid cultures (**Figures 3B, D** **)**. Conversely, *NRN1* overexpression in PDCs significantly upregulated *NRN1* mRNA levels compared with transfection with control vector (**Figures 3E, G** **)** and increased the viabilities of RCC-PDC spheroid cultures (**Figures 3F, H** **)**.

We examined whether *NRN1* expression modulates *CXCR4* expression in RCC-PDC spheroid cultures. *NRN1* silencing (**Figures 3I, J** **)** and overexpression (**Figures 3K, L** **)** significantly downregulated and upregulated *CXCR4* expression, respectively. Moreover, *CXCR4*-specific siRNAs (siCXCR4 #1 and #2) significantly repressed *CXCR4* expression levels compared with siControl (**Figures 3M, O** **)** and impaired the viabilities of RCC-PDC spheroid cultures (**Figures 3N, P** **)**. Additionally, we evaluated the correlation between NRN1 and CXCR4 immunoreactivities in our 100 ccRCC cases. Of 25 high CXCR4 immunoreactivity cases, 12 (48%) and 13 (52%) had high and low NRN1 immunoreactivities, respectively, and of 75 low CXCR4 immunoreactivity cases, 9 (12%) and 66 (88%) had high and low NRN1 immunoreactivities, respectively (*P* = 1.0e-4 by Chi-square test).

As a potential NRN1-activated transcriptional factor, nuclear factor of activated T-cells (NFAT) c4 may activate CXCR4 transcription in ccRCC based on previous literature describing that NRN1 activates NFATc4 *via* binding to insulin receptor in neuronal cells (19), and that NFATc4-binding on the CXCR4 promoter increases CXCR4 expression in 3D spheroid culture of ovarian cancer cells (20). In this context, it is notable that *NFATC4* mRNA expression is substantially correlated with *NRN1* expression in TCGA PanCancer Atlas RNA-seq dataset of ccRCC cohort (Spearman’s correlation coefficient = 0.333, q-value 1.38e-10). Moreover, qRT-PCR experiment demonstrated that NRN1 knockdown decreased *NFATC4* mRNA levels in both RCC-PDC1 and 2, supporting the notion of intermediate function of NFATC4 between NRN1 and CXCR4 (**Supplementary Figure 6**).

### NRN1 Silencing Is a Potential Therapeutic Strategy for RCC Tumor

**Figure 4:**
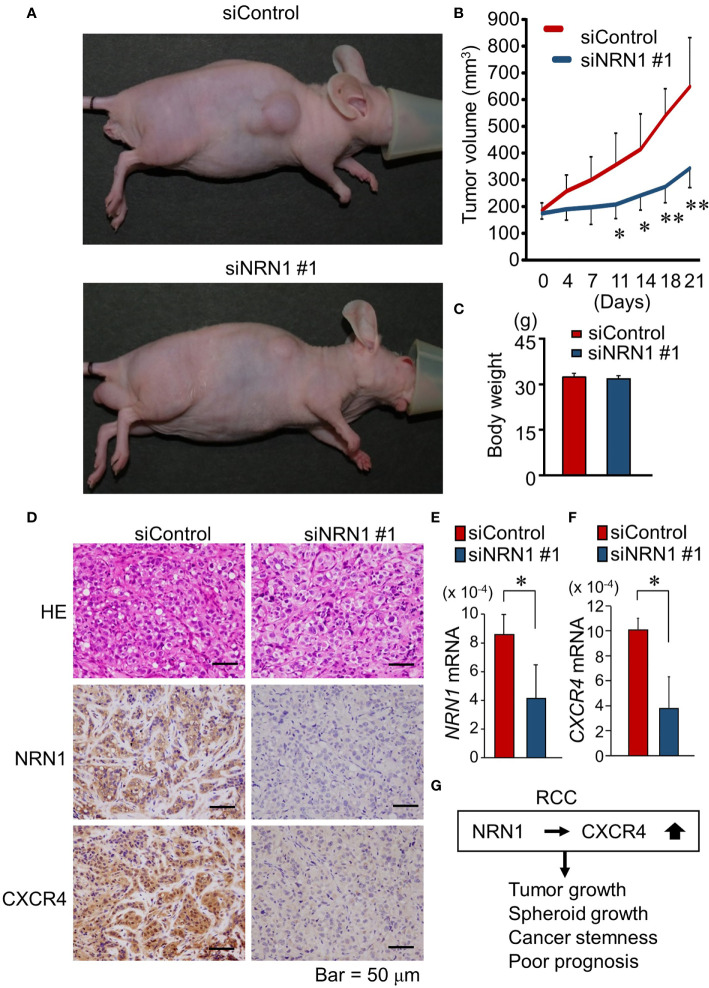
NRN1 silencing suppresses *in vivo* growth of RCC-PDC-derived xenograft tumors. **(A)** Representative images of xenograft tumor-bearing nude mice at time of sacrifice. **(B)** Volume of xenograft tumors derived from RCC-PDC1 cells treated with siControl (*n* = 5, red) or siNRN1 #1 (*n* = 5, blue). **(C)** Body weights of mice at time of sacrifice. **(D)** Representative images of hematoxylin and eosin (HE) staining and NRN1 and CXCR4 IHC staining in dissected xenograft tumors treated with siControl or siNRN1 #1. Scale bars, 50 μm. **(E, F)** *NRN1* **(E)** and *CXCR4* **(F)** levels in xenograft tumors analyzed by qRT-PCR. Data are shown as mean ± SD, *n* = 5; *P < 0.05, **P < 0.01 by two-sided Student’s *t*-test. **(G)** Schematic representation of oncogenic function of NRN1 and CXCR4 in RCC tumors.

We further questioned whether NRN1-specific siRNAs can repress *in vivo* RCC tumor growth. In RCC-PDC1-derived xenograft models in nude mice, siNRN1 #1 or siControl was directly injected into the subcutaneous tumors twice a week when the tumor volume exceeded 150 mm^3^. siNRN1 injection significantly repressed the growth of RCC-PDC1-derived tumors compared with siControl injection (**Figures 4A, B** **)**, while body weights of mice were not substantially different between the 2 groups (**Figure 4C**). In the dissected tumors of siNRN1 #1-treated mice, NRN1 and CXCR4 immunoreactivities were negligible and *NRN1* and *CXCR4* mRNA levels were significantly decreased compared with those of siControl-treated mice (**Figures 4D–F**). In summary, NRN1 plays an oncogenic role in RCC in cooperation with CXCR4 (**Figure 4G**).

## Discussion

In the present study, we identified that NRN1 is as a poor prognostic biomarker for RCC. In PDC/PDCX models, we showed that NRN1 promotes *in vitro* and *in vivo* RCC proliferation, and *NRN1* silencing downregulates *CXCR4*, a gene co-expressed with *NRN1*, that is also a prognostic and CSC marker in RCC. Our data suggest that NRN1 exhibits a tumor-promoting effect in RCC in collaboration with CXCR4.

We have previously showed that NRN1 is abundantly expressed in testicular germ cell tumor PDC spheroid culture, and NRN1 expression is transcriptionally regulated by hypoxia inducible factor 1α (HIF1α) (12). Intriguingly, hypoxia-induced expression of NRN1 was also shown in melanoma cells, and soluble NRN1 induced to form vascular mimicry by melanoma cells (21). In ccRCC tumors, the loss-of-function mutations of von Hippel–Lindau tumor suppressor (VHL), an E3 ubiquitin ligase that targets HIF1α under normoxic conditions, are usually responsible for hypoxia response activation (22, 23). Hypoxic conditions in RCC can be also modulated by obesity, which often leads to the accumulation of peritumoral adipose tissue (24). It is notable that CXCR4 is a prototypic HIF1α target gene in RCC (22), and also identified as a CSC marker in RCC (7). Overall, both NRN1 and CXCR4 expression can be modulated by hypoxic environments in RCC, while a molecular mechanism how NRN1 modulates CXCR4 expression remains to be clarified. Because NRN1 activates transcription factor nuclear factor of activated T-cells (NFAT) c4 *via* the binding NRN1 to insulin receptor and the activation of calcium signaling-calmodulin-calcineurin axis in neuronal cells (19), NRN1 may also activate CXCR4 transcription by modulating some transcriptional machinery in RCC cells.

In terms of NRN1 functions, previous gain- and loss-of-function studies have indicated that NRN1 stimulates anchorage-independent growth and tumorigenesis, suggesting potential NRN1-mediated transformation ability (25). In addition, NRN1 has been reported to have a protective effect on cortical progenitor cells in the fetal brain by preventing caspase-dependent apoptosis (26). NRN1 has been also reported to be involved in cell-cell and cell-ECM adhesion (27). Notably, our pathway analysis revealed that ECM-associated pathways were enriched among genes correlated with both NRN1 and CXCR4 expression in TCGA ccRCC RNA-seq dataset. Furthermore, our pathological analysis demonstrated that NRN1 high immunoreactivity tended to correlated with lymph node metastasis and distant metastasis. These findings suggest that NRN1 may also facilitate to develop metastasis by modulating the ECM of RCC tumor cells or surrounding stromal cells. Notably, soluble NRN1 promotes the migration of melanocyte cells and higher serum NRN1 levels were observed in melanoma patients compared with healthy donors, indicating that soluble NRN1 may also be a potential diagnostic biomarker in RCC patients (21).

In our clinicopathological analysis, high NRN1 immunoreactivity was significantly correlated with high serum corrected calcium levels. Dysregulation of Ca^2+^ signaling has been found as one of the key features of RCC progression. Epidemiologically, hypercalcemia has been shown to be a factor for poor prognosis in RCC (28, 29). In addition, Ca^2+^ signaling/influx in RCC cells has been shown to play a tumor promoting role (30, 31). Interestingly, in mouse neuronal cells, *NRN1* is stimulated by Ca^2+^ influx and also upregulates intracellular Ca2^2+^ concentration and signaling through potassium channel activation (19, 32). Besides, Ca^2+^ signaling is associated with CXCR4 regulation. Ca^2+^-induced CXCR4 expression is found in bone marrow cells in mice (33) and CXCR4-mediated intracellular Ca^2+^ upregulation is implicated in cell migration in breast cancer and oral squamous cell carcinoma (34, 35). Therefore, NRN1 and CXCR4 may be associated with perturbation of calcium regulation in RCC.

NRN1 immunoreactivity was also correlated with serum CRP levels in our study. NRN1 has been reported as an angiogenic factor in tumors (36), and CRP, a marker of inflammation, is known as an informative predictor for patient survival in RCC (37). In addition, CXCR4 plays an important role in inflammation with its ligand CXCL12 and associates with pathological processes such as tumor proliferation, angiogenesis, and metastasis (38). These findings suggest that NRN1 and CXCR4 may coordinately contribute to tumor inflammation and angiogenesis (39).

PDC/PDX models are useful tools for investigating molecular mechanisms that can be applied to personalized medicine and drug screening. In particular, advanced ccRCC is usually treated by targeted therapies or combination treatment including VEGF-TKIs, mTOR inhibitors, and immunotherapy, so the PDC/PDX models may exert advantages to investigate drug sensitivity and resistance against such treatments. Considering that NRN1 is an angiogenic factor in literature, we could speculate that NRN1 may have relevance in VEGF-TKI resistance. We thus consider that the generation of NRN1-overexpressed or -silenced PDC models from both ccRCC and non-ccRCC will address the point whether NRN1 contributes to the mechanism of VEGF-TKI resistance.

We used patient-derived ccRCC cells in the present study as NRN1 is abundantly expressed in these models, although we understand the usefulness of cell line-based study as the molecular mechanisms of established cell lines have been well characterized compared with our patient-derived models. While we successfully showed the oncogenic relevance of DPP4 in both patient-derived RCC spheroid cultures and RCC cell lines (10), RCC cell line such as 786-O may also be useful to characterize the biological function and potential regulation roles of NRN1.

Taken together, the present study suggests that NRN1 can be a potential diagnostic and therapeutic target in RCC.

## Conclusions

NRN1 regulates RCC proliferation in cooperation with CXCR4. Patient-derived cancer models would be useful for elucidating novel therapeutic targets in RCC.

## Data Availability Statement

The original contributions presented in the study are included in the article/**Supplementary Material**. Further inquiries can be directed to the corresponding authors.

## Ethics Statement

The studies involving human participants were reviewed and approved by Institutional Review Board of the Saitama Medical Center, Saitama Medical University. The patients/participants provided their written informed consent to participate in this study. The animal study was reviewed and approved by Animal Care and Use Committee of Saitama Medical University.

## Author Contributions

SKam, KH, and SI: study concepts and design. SKam, TS, WS, SKit, and KI: data acquisition. SKam, TS, and KI: quality control of data and algorithms. SKam, TS, and KI: data analysis and interpretation. SKam, TS, and KI: statistical analysis. SKam, KI, and KH: manuscript preparation. KH, TS, TI, SKaw, and SI: manuscript editing/review. All authors read and approved the final manuscript.

## Funding

This study was supported by the Grants-in-Aid for Scientific Research from the Japan Society for the Promotion of Science (20K21667, 20H03734, 20K21636, and 21H02981) and by the Grant for Cancer Research from the Vehicle Racing Commemorative Foundation.

## Conflict of Interest

The authors declare that the research was conducted in the absence of any commercial of financial relationships that could be construed as a potential conflict of interest.

## Publisher’s Note

All claims expressed in this article are solely those of the authors and do not necessarily represent those of their affiliated organizations, or those of the publisher, the editors and the reviewers. Any product that may be evaluated in this article, or claim that may be made by its manufacturer, is not guaranteed or endorsed by the publisher.
