# Supplementary material for: Clinicopathological and Preclinical Patient-Derived Model Studies Define High Expression of NRN1 as a Diagnostic and Therapeutic Target for Clear Cell Renal Cell Carcinoma
Source: Front Oncol. 2021 Nov 3;11:758503. doi: 10.3389/fonc.2021.758503 (PMC8595331; doi:10.3389/fonc.2021.758503)
Supplement: Supplementary file 1 [file DataSheet_1.pdf]

Supplementary Table 1 | Characteristics of patients for RCC-PDC1 and RCC-PDC2.

Cancer cells	Age	Gender	Stage	Pathological findings	Grade	Infiltration	Surgical procedure
RCC-PDC1	46	M	T3bN0M1 Stage IV	Clear cell renal cell carcinoma	3	b	Radical nephrectomy
RCC-PDC2	80	M	T1bN0M0 Stage I	Clear cell renal cell carcinoma	2	b	Radical nephrectomy

Supplementary Table 2 | Sequences of primers for quantitative reverse transcription polymerase chain reaction (qRT-PCR).

Primer name	Sequence (5' to 3')
*NRN1*_Fw	GGGCGACAGCATGGCCAACT
*NRN1*_Rv	CCGCTGCCGCAGAGTTCGAA
*CXCR4*_Fw	GCATGACGGACAAGTACAGGCT
*CXCR4*_Rv	AAAGTACCAGTTTGCCACGGC
*ACTB*_Fw	CCTGGCACCCAGCACAA
*ACTB*_Rv	AGCGAGGCCAGGATGGA
*NFATC4*_Fw	CCGTATCACAGGCAAGATGGT
*NFATC4*_Rv	AGCAGAGTCATCTCCAACACCTT

Supplementary Table 3 | Sequences of short interfering RNAs (siRNAs).

siRNA		Sequence (5’ to 3’)
siNRN1 #1	Sense strand	AUUCUUUUCUCAGUUUAUCCC
	Antisense strand	GAUAAACUGAGAAAAGAAUCC
siNRN1 #2	Sense strand	AAAUAAAACACAAGAAAUCAA
	Antisense strand	GAUUUCUUGUGUUUUAUUUGC
siCXCR4 #1	Sense strand	UGAAAUUAGCAUUUUCUUCAC
	Antisense strand	GAAGAAAAUGCUAAUUUCAAU
siCXCR4 #2	Sense strand	CCGACCUCCUCUUUGUCAUCA
	Antisense strand	AUGACAAAGAGGAGGUCGGCC
siControl	Sense strand	GUACCGCACGUCAUUCGUAUC
	Antisense strand	UACGAAUGACGUGCGGUACGU

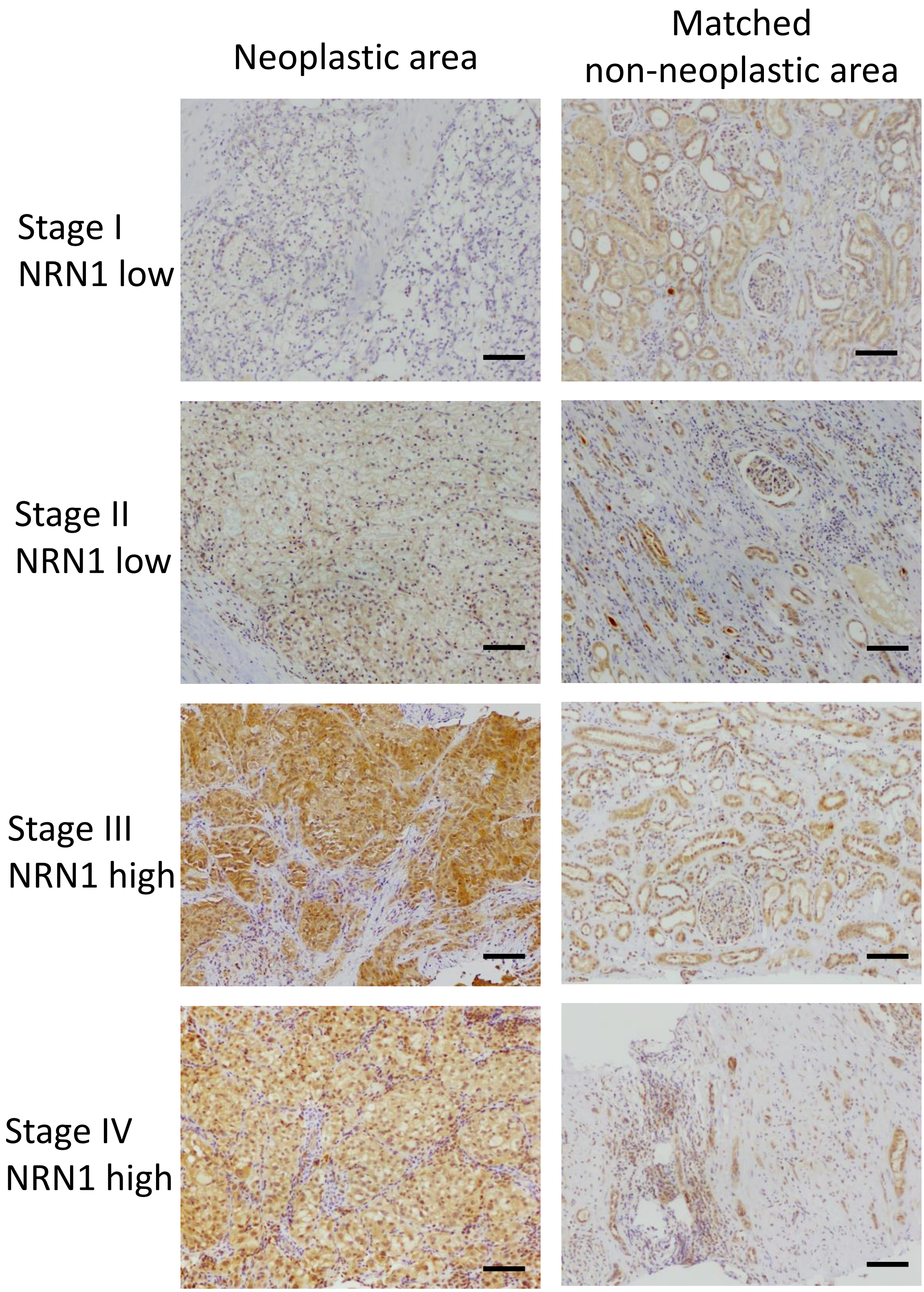

Supplementary Figure 1 | Representative NRN1 immunohistochemistry staining of neoplastic area (left panel) and its paired corresponding non-neoplastic area (right panel) with each stage in ccRCC tissue sections. Scale bars, 100 µm.

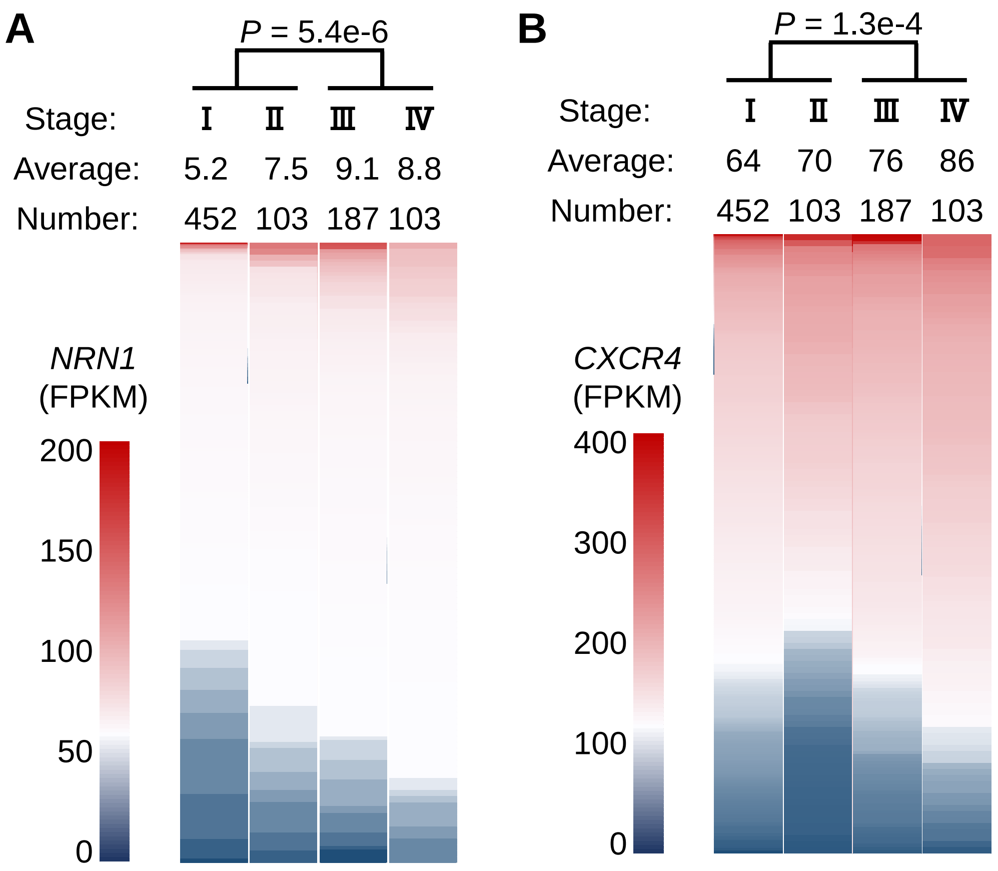

Supplementary Figure 2 | Expression levels of *NRN1* and *CXCR4* associated with clinical tumor stages in RCC. **(A, B)** Association of *NRN1* (A) and *CXCR4* (B) mRNA expression levels and clinical tumor stages in RCC. Expression levels are shown in Fragments Per Kilobase of exon per Million mapped reads (FPKM). Data are retrieved from The Human Protein Atlas (https://www.proteinatlas.org/).

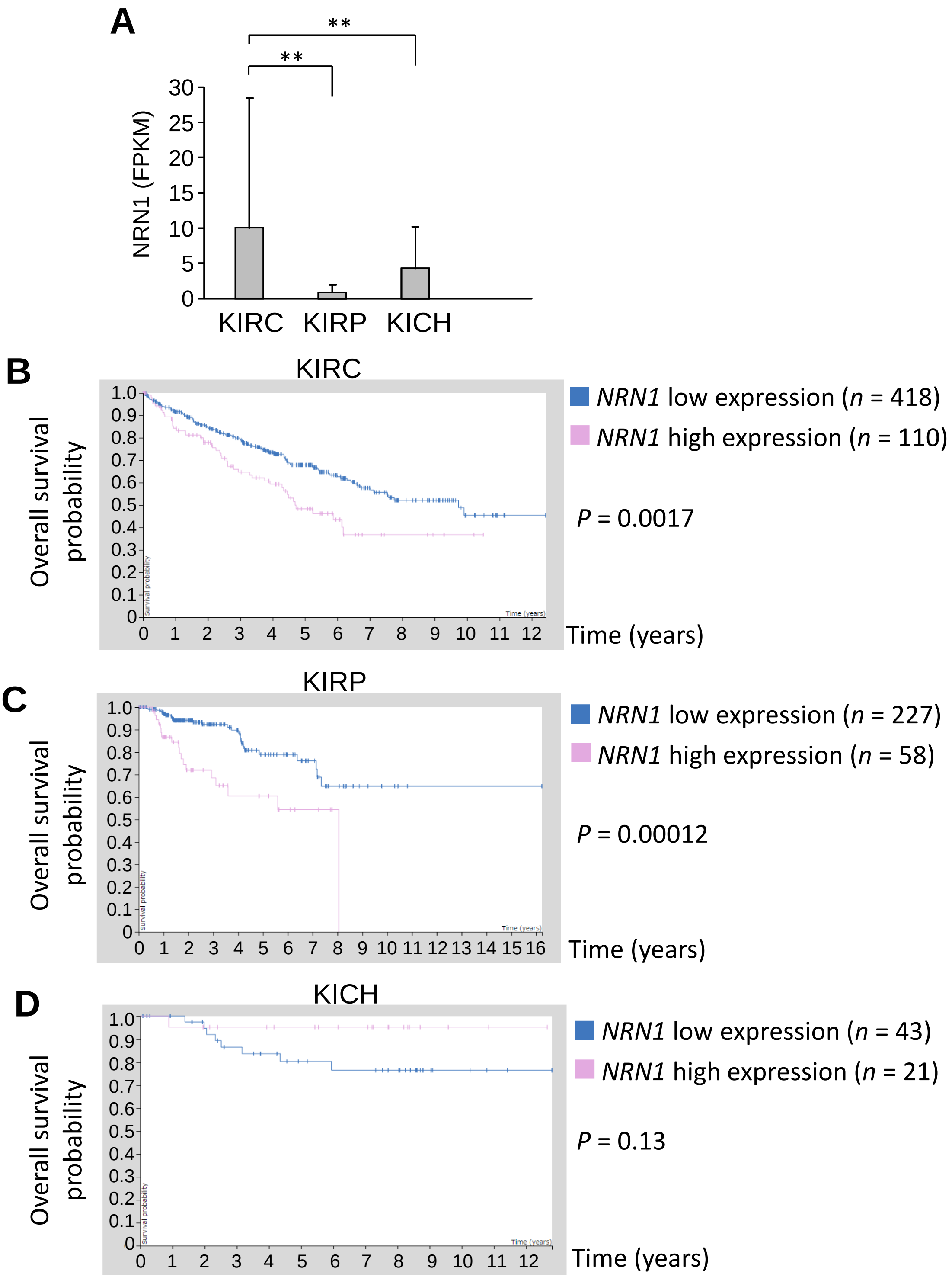

Supplementary Figure 3 | *NRN1* is associated with prognosis in patients with KIRC and KIRP. **(A-D)** Data of *NRN1* expression and patient overall survival were retrieved from TCGA dataset (ccRCC [KIRC], papillary RCC [KIRP], and chromophobe RCC [KICH]) in The Human Protein Atlas. *NRN1* expression levels are shown in FPKM (A). Overall survival of patients with KIRC (B), KIRP (C), and KICH (D) were analyzed using Kaplan-Meier method with stratification by *NRN1* expression levels. Statistical significance was evaluated by log-rank test.

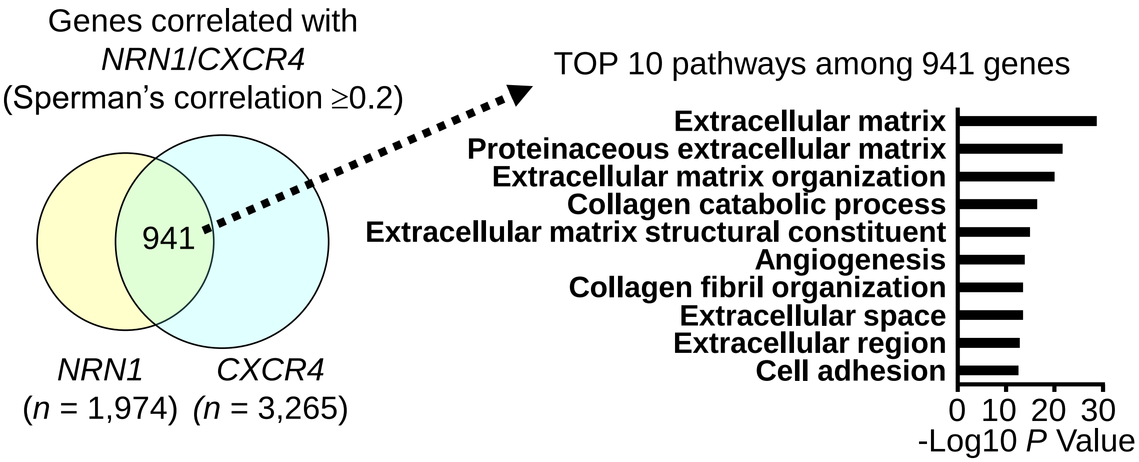

Supplementary Figure 4 | Gene ontology analysis of co-expressed genes with both *NRN1* and *CXCR4*. Expression data retrieved from cBioPortal (https://www.cbioportal.org) were analyzed for mRNA co-expression and the selected 941 common genes were further analyzed with gene ontology using the Database for Annotation, Visualization and Integrated Discovery (DAVID) (https://david.ncifcrf.gov/tools.jsp).

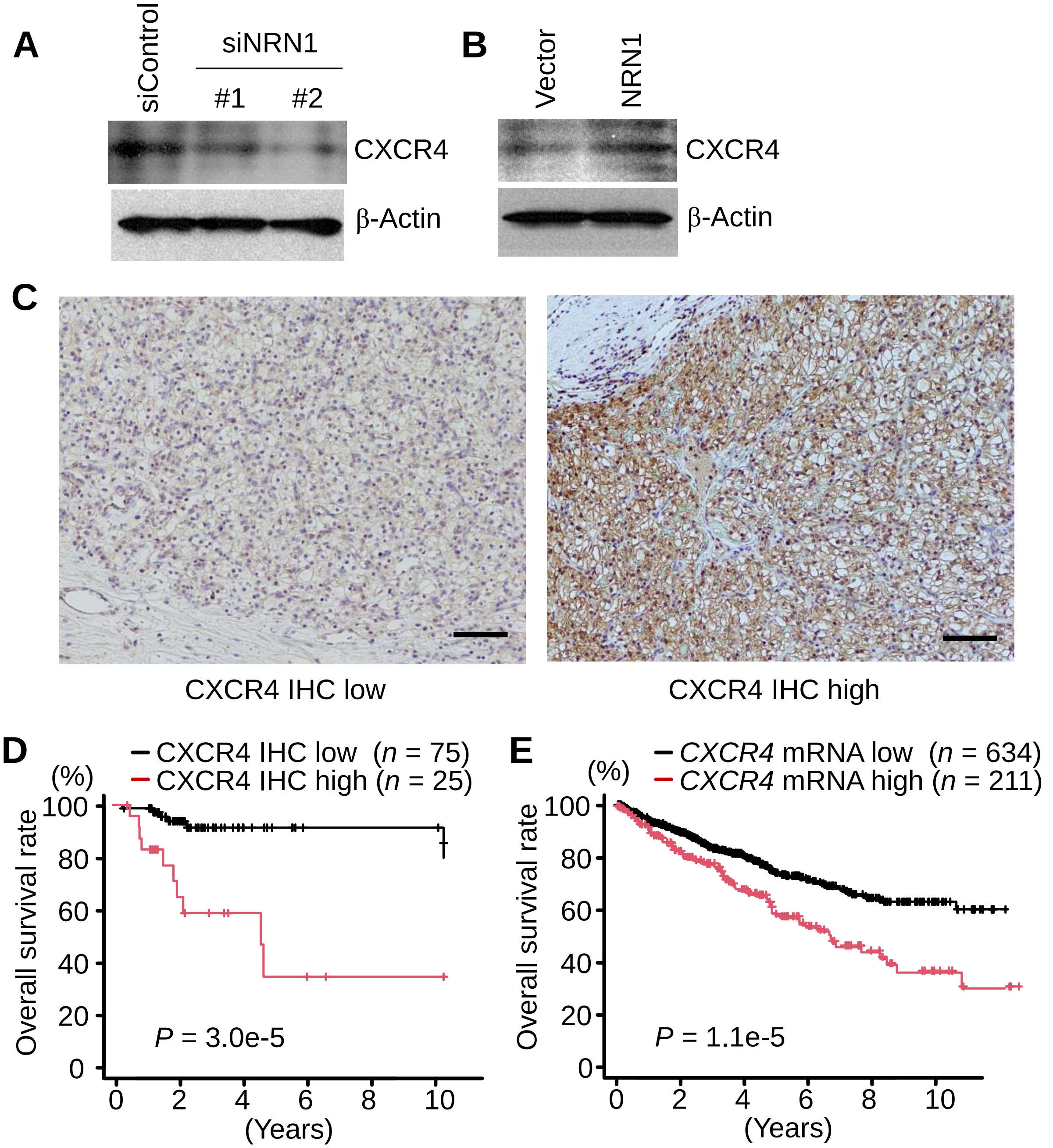

Supplementary Figure 5 | CXCR4 is associated with poor prognosis in RCC patients. **(A, B)** RCC-PDC1 cells were transfected with siRNAs (A) or expression vectors (B) indicated, and then subjected to Western blot analysis using CXCR4 or β-actin antibodies. **(C)** Representative immunohistochemistry (IHC) staining of low (left panel) and high (right panel) expression of CXCR4 in RCC tissue sections. Scale bars, 100 µm. **(D)** Overall survival of 100 clear cell RCC (ccRCC) patients with low or high CXCR4 IHC staining was analyzed by Kaplan-Meier method. Statistical significance was evaluated by log-rank test. **(E)** *CXCR4* expression data in TCGA RCC dataset were retrieved from The Human Protein Atlas (*n* = 845). Overall survival of RCC patients with low or high *CXCR4* expression levels were analyzed by Kaplan-Meier method. High and low *CXCR4* expressions were determined at an expression cut off level as 99.5 FPKM (expression level range: 2.1-372.0, expression median level: 61.8 FPKM, *P* = 1.1e-5). Statistical significance was evaluated by log-rank test.

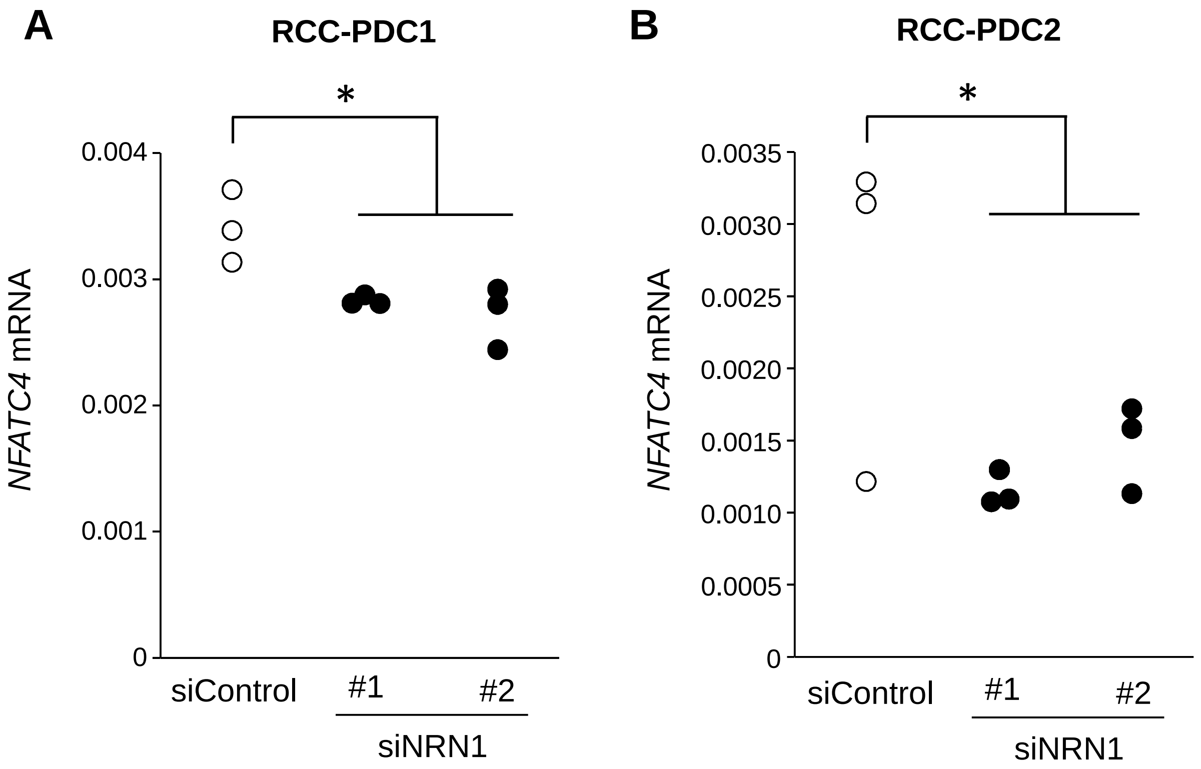

Supplementary Figure 6 | NRN1 silencing represses *NFATC4* mRNA in RCC-PDCs. **(A, B)** RCC-PDC1 (A) and 2 (B) spheroid cultures were transfected with NRN1 (siNRN1 #1 and #2) or control (siControl) siRNAs. *NFATC4* mRNA levels were analyzed by qRT-PCR (n = 3. Data are shown as means ± SD. * $P < 0.05$ by two-way ANOVA test.
